# Dual Challenges in the Context of Healthy Aging: A Comprehensive Exploration of the Association between Malnutrition and Cognitive Decline in Disabled Elderly

**DOI:** 10.14336/AD.2025.0337

**Published:** 2025-04-02

**Authors:** Runyuan Yu, Lixia Wang, Yifan Liu, Yimeng Hu, Zuncheng Zheng, Xiaoyu Wang, Yuexia Chen, Yulian Liu

**Affiliations:** ^1^Department of Rehabilitation Medicine, The Affiliated Taian City Central Hospital of Qingdao University, Taian, 271000, China.; ^2^Gaotang County Center for Disease Control and Prevention, Liaocheng, 252800, China.; ^3^Binzhou Medical University, Binzhou, 264003, China.; ^4^Department of Skills Training Center, The Affiliated Taian City Central Hospital of Qingdao University, Taian, 271000, China.

**Keywords:** disabled elderly, malnutrition, cognitive decline, biological mechanisms, nutritional intervention

## Abstract

Disabled older adults represent a population requiring special attention in the context of global aging, with malnutrition and cognitive decline being prevalent and interrelated health concerns. This review systematically examines the association between malnutrition and mental deterioration in this population, with an in-depth exploration of the potential biological mechanisms underlying this relationship. Current evidence suggests that malnutrition accelerates cognitive decline through multiple pathways, including neurotransmitter synthesis impairment, insufficient cerebral energy supply, chronic inflammation and oxidative stress, blood-brain barrier dysfunction, and reduced neuroplasticity. Additionally, dysregulation of the gut-brain axis, an emerging mechanism, may influence brain health via alterations in the gut microbiota. This review aims to provide a theoretical foundation for understanding the intricate relationship between malnutrition and cognitive impairment while offering insights into optimizing health management and nutritional strategies for disabled older adults.

## 1. Introduction

With the accelerating global trend of population aging, health issues related to the elderly have become a focal point of public health and social concern. According to United Nations projections, by 2050, the global population of individuals aged 65 and above will reach 1.6 billion, accounting for approximately 16% of the world population [1]. Among the elderly population, disabled older adults have become an essential subject of research and attention. Due to limitations in physical or cognitive functions, disabled, elderly individuals are unable to live independently in daily activities and often require long-term care. This poses significant challenges to individuals, families, and social healthcare resources [2]. Among the disabled elderly, malnutrition is a common and severe health issue, often caused by insufficient food intake, absorption disorders, or metabolic abnormalities [3]. Studies indicate that the prevalence of malnutrition in disabled elderly individuals is significantly higher than in the general elderly population, reaching 30% to 50% [4,5]. Malnutrition not only affects the physical function of elderly individuals but may also accelerate cognitive decline, further worsening their health status and increasing the degree of disability [6].

Cognitive decline is another essential health issue closely related to the quality of life in the elderly. Cognitive decline not only manifests as a reduction in abilities such as memory, language, and attention but can also develop into more severe conditions like dementia (e.g., Alzheimer's disease (AD)) [7]. Research indicates that the prevalence of cognitive decline among elderly individuals aged 65 and above is about 10%-20%, with higher rates in the older population [8,9]. For disabled, elderly individuals, cognitive decline and malnutrition may form a vicious cycle: malnutrition causes brain function damage, further accelerating cognitive degeneration; conversely, cognitive decline may lead to abnormal eating behaviors, increasing the risk of malnutrition [10]. In this context, investigating the relationship between malnutrition and cognitive decline in disabled elderly individuals is of great practical significance. Studying their association and potential mechanisms can provide scientific evidence for the early identification of high-risk groups and formulation comprehensive intervention strategies. This not only helps improve the health status and quality of life of disabled and elderly individuals but also guides the reduction of the caregiving burden on families and society.

## 2. The Association Between Malnutrition and Cognitive Decline

In recent years, the close association between malnutrition and cognitive decline has gradually become a focal point of academic research. Studies show that the nutritional status of the elderly is an essential indicator of their overall health and a crucial factor in maintaining normal cognitive function. Han et al. [11] conducted a cross-sectional study on community elderly individuals aged 75 and above. They found that 55.7% of the subjects exhibited physical frailty, with the degree of frailty significantly negatively correlated with cognitive function. Among these, malnutrition was identified as a significant risk factor for frailty and cognitive impairment. Song et al. [12] were the first to link elderly dietary patterns (the dietary pyramid) with cognitive function, discovering that higher adherence to this dietary pattern was associated with better cognitive abilities. Moreover, Pizzol et al. [13] demonstrated that malnutrition significantly increases the risk of cognitive impairment and is highly correlated with brain developmental disorders, emphasizing the global importance of strengthening nutritional interventions for the elderly.

**Figure 1.**
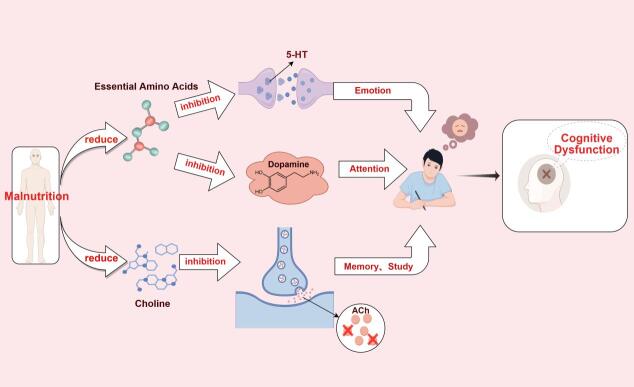
Malnutrition affects cognitive function through neurotransmitter synthesis pathways.

These studies collectively reveal the close relationship between malnutrition and cognitive decline. However, due to the high heterogeneity in the health status of the elderly population, the specific impact of malnutrition on cognitive function may vary significantly. Therefore, further exploration of how different types of malnutrition affect cognitive function through various mechanisms is of great importance for developing effective intervention strategies.

## 3. Mechanisms of Malnutrition’s Impact on Cognitive Function

Malnutrition affects cognitive function through multiple biological pathways, including neurotransmitter synthesis disorders, insufficient brain energy supply, chronic inflammation, and oxidative stress. These mechanisms intertwine and collectively accelerate cognitive decline.

### 3.1 Impairment of Neurotransmitter Synthesis

Neurotransmitters play a central role in maintaining cognitive function, and their proper synthesis depends on adequate nutrient intake [14,15]. Malnutrition can impair the synthesis and function of neurotransmitters. It can lead to an insufficient supply of essential amino acids (such as tryptophan and tyrosine), thereby inhibiting the production of serotonin and dopamine. The decrease in these neurotransmitter levels is closely associated with memory loss and a decline in learning abilities [16,17].

Moreover, malnutrition can reduce choline intake, a precursor of acetylcholine [18]. As one of the primary central neurotransmitters, acetylcholine plays a crucial role in regulating memory and attention. A deficiency in acetylcholine levels may significantly impair cognitive function. Choline deficiency is also associated with metabolic disorders, which can further exacerbate cognitive decline through systemic health deterioration [19] (according to Fig. 1).

### 3.2 Insufficient Brain Energy Supply

The brain is one of the most metabolically demanding organs in the human body, and its function relies on a sufficient and stable energy supply. Malnutrition weakens brain energy metabolism through various pathways, significantly impacting cognitive function [20]. Glucose metabolism disorder is one of the core mechanisms behind malnutrition and insufficient brain energy supply. Glucose is the primary energy source for brain cells, and its metabolic disruption directly affects neuronal function. Under malnourished conditions, hypoglycemia or insulin resistance reduces glucose utilization efficiency, leading to synaptic transmission dysfunction and neural network imbalance [21]. Malnutrition-induced insulin signaling disruption is closely associated with metabolic diseases (such as type 2 diabetes), which are important risk factors for cognitive impairment [22].

**Figure 2.**
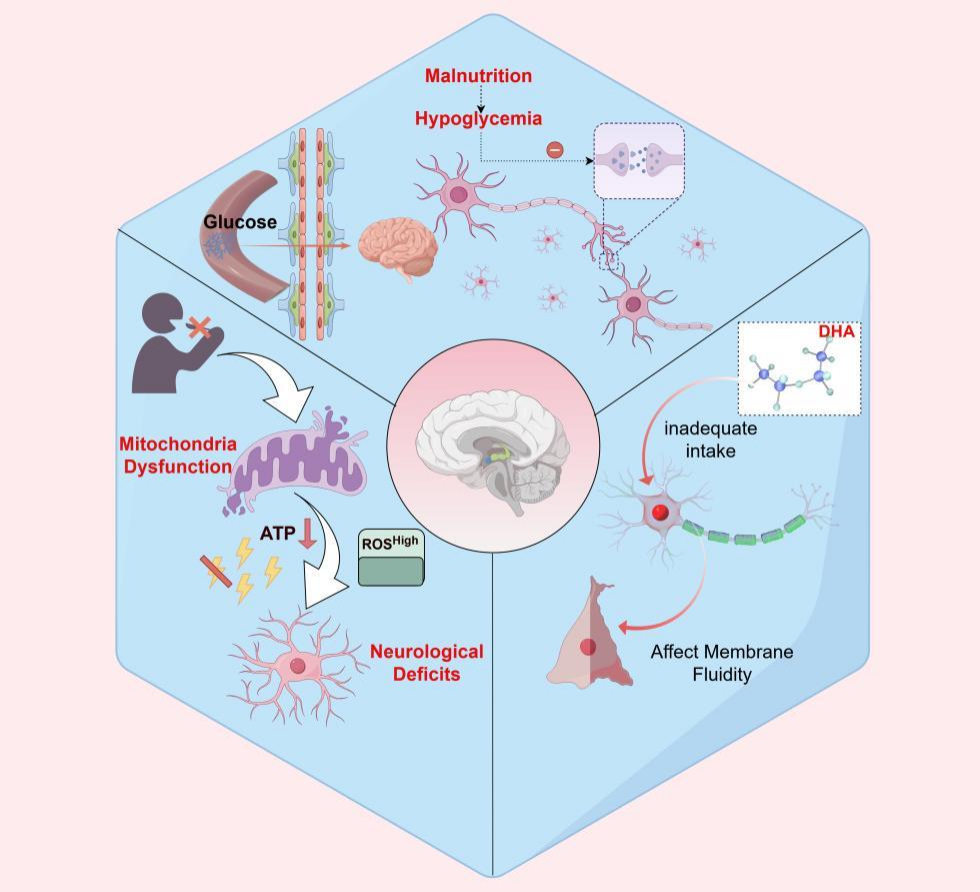
Malnutrition affects cognitive function through the brain's energy supply pathways.

Mitochondrial dysfunction is another critical pathway. Malnutrition affects mitochondrial oxidative phosphorylation by causing deficiencies in essential micronutrients (such as vitamins B1, B6, B12, iron, and copper), weakening Adenosine triphosphate (ATP) production capacity [23]. The hippocampus and other brain regions with high energy demands are mainly dependent on proper mitochondrial function [24]. Studies have shown that mitochondrial dysfunction impairs neuronal energy supply and increases free radical production, exacerbating oxidative stress and further damaging neuronal structure and function [25].

Additionally, insufficient intake of essential fatty acids negatively impacts the brain's energy supply. Long-chain polyunsaturated fatty acids play vital roles in maintaining neuronal membrane fluidity, promoting metabolite transport, and optimizing energy utilization [26]. Research indicates that a dietary deficiency of Long-chain polyunsaturated fatty acids compromises synaptic structure integrity, leading to decreased efficiency in glucose and oxygen metabolism in the brain [27]. (according to Fig. 2)

### 3.3 Chronic Inflammation and Oxidative Stress

Chronic inflammation and oxidative stress are key mechanisms through which malnutrition affects cognitive function. These two factors interact and collectively accelerate neuronal damage and cognitive decline.

Malnutrition alters immune and metabolic states, leading to systemic chronic inflammatory responses. Studies have shown that levels of pro-inflammatory factors (such as Interleukin-6 [IL-6], Tumour necrosis factor-alpha [TNF-α], and C-reactive protein [CRP]) are significantly elevated in malnourished individuals. These factors can cross the blood-brain barrier and induce neuroinflammation in the brain [28,29]. Neuroinflammation results in the overactivation of microglia, which release more pro-inflammatory cytokines, thereby damaging neuronal structure and function [30]. Moreover, a pro-inflammatory state is closely associated with decreased synaptic plasticity in the brain, which weakens learning and memory functions [31].

**Figure 3.**
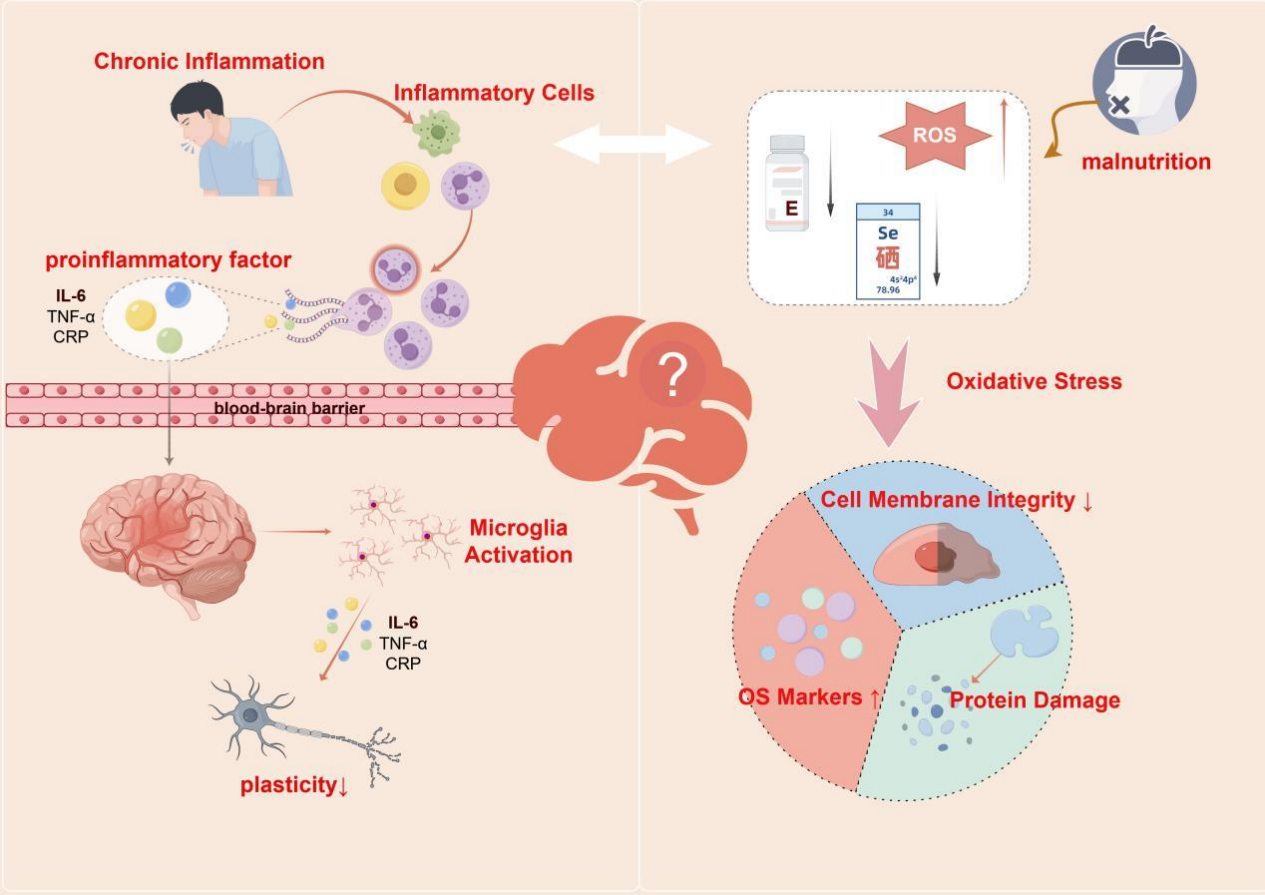
Malnutrition affects cognitive function through chronic inflammation and oxidative stress pathways.

Malnutrition is often accompanied by a decline in antioxidant capacity, leading to increased production of free radicals (such as Reactive oxygen species [ROS] and Reactive nitrogen species [RNS]) [32]. The accumulation of free radicals damages neuronal cell membranes and mitochondrial function, triggering lipid peroxidation and protein damage [33]. Under malnourished conditions, deficiencies in antioxidants (such as vitamins E, C, and selenium) further exacerbate oxidative stress, as indicated by elevated oxidative stress markers (such as Malondialdehyde [MDA]) and pro-inflammatory factor CRP. This ultimately compromises the integrity of neuronal cell membranes and worsens cognitive impairment [34,35]. For example, increased levels of oxidative stress markers (such as MDA and 8-hydroxy-2'-deoxyguanosine [8-OHdG]) have been confirmed to be positively correlated with cognitive decline [36].

Additionally, chronic inflammation increases free radical production while suppressing antioxidant pathways, thereby exacerbating oxidative stress reactions [37]. At the same time, oxidative stress can activate the NF-κB signaling pathway and other inflammation-related pathways, further intensifying the inflammatory response and creating a vicious cycle [38]. This synergistic effect accelerates neuronal damage and synaptic degeneration, ultimately leading to cognitive decline (according to Fig. 3).

### 3.4 Impaired Blood-Brain Barrier Function

The integrity of the Blood-brain barrier (BBB) is crucial for maintaining brain homeostasis and neural function. Its primary role is to regulate the transport of nutrients and metabolic byproducts through highly selective permeability while preventing toxic substances from entering brain tissue [39]. Studies have shown a causal relationship between increased BBB permeability and significant declines in attention, memory, and executive function, suggesting that BBB integrity is a fundamental biological basis for maintaining cognitive function [40]. One of the primary mechanisms by which malnutrition impairs BBB function is endothelial dysfunction. A deficiency in essential fatty acids, such as omega-3 polyunsaturated fatty acids, weakens the integrity of vascular endothelial cells and reduces the expression of tight junction proteins (such as claudin-5 and occludin) [41]. Tight junction proteins are critical for maintaining the selective permeability of the BBB, and their reduced expression significantly increases BBB permeability. This allows harmful substances, such as inflammatory cytokines and free radicals, to enter brain tissue, triggering neuroinflammation and oxidative stress [42]. Additionally, oxidative stress induced by malnutrition can activate Matrix metalloproteinases (MMPs), which degrade tight junction proteins, further increasing BBB permeability and accelerating cognitive impairment [43] (according to Fig. 3).

Another critical factor is the reduced levels of carrier proteins. In a malnourished state, plasma levels of albumin and other carrier proteins (such as transferrin) decrease. This weakens the BBB's ability to clear metabolic waste and toxic molecules and leads to insufficient nutrient supply (such as glucose and essential amino acids), further impairing brain energy metabolism and neurotransmitter synthesis [44].

### 3.5 Decline in Neural Plasticity

Neural plasticity refers to the brain's ability to self-adjust and reorganize structurally and functionally in response to internal and external environmental stimuli. It forms the basis for learning and memory [45]. Maintaining neural plasticity requires adequate energy supply, balanced neurotransmitter levels, and a well-functioning neuronal microenvironment. Malnutrition weakens neural plasticity through various pathways, significantly impacting cognitive function.

Impaired synaptic plasticity is one of the core mechanisms by which malnutrition affects neural plasticity. Synaptic plasticity is primarily regulated by Brain-derived neurotrophic factor (BDNF), which plays a crucial role in neuronal survival, synapse formation, and functional regulation [46]. In a malnourished state, the expression of BDNF significantly decreases, possibly weakening synaptic stability and plasticity by inhibiting the expression of synaptic-related proteins (such as synapsin and PSD-95) [47]. Studies have shown that deficiencies in micronutrients such as zinc, iron, vitamin D, and unsaturated fatty acids are closely related to reduced BDNF levels, leading to a decline in learning and memory function [48].

**Figure 4.**
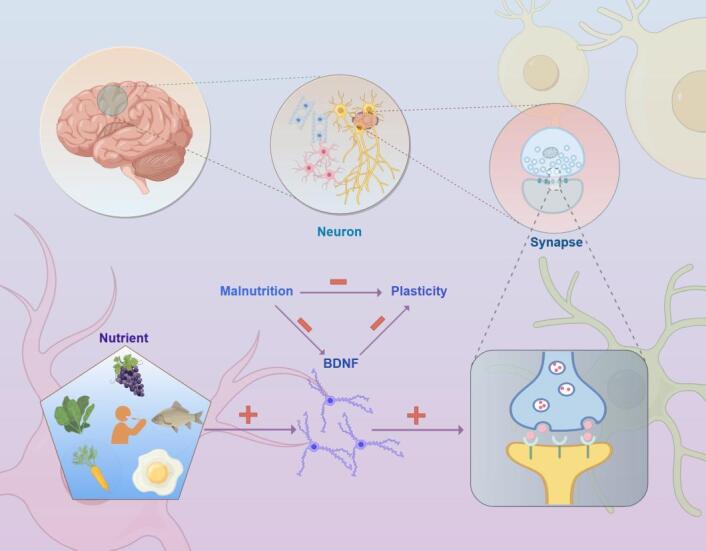
Malnutrition affects cognitive function through neuroplastic pathways.

A reduced ability for synaptic remodeling is another manifestation of decreased neural plasticity. Malnutrition can impair the synthesis of neurotransmitters (such as acetylcholine, serotonin, and dopamine) and inhibit the supply of key nutrients (such as choline and tryptophan), thereby weakening the efficiency of information transmission between synapses [49,50]. A diminished ability for synaptic remodeling further limits the brain's adaptive changes in response to external stimuli, leading to decreased learning efficiency and weakened memory capacity (according to Fig. 4).

### 3.6 Insufficient Cerebral Blood Flow

Insufficient cerebral blood flow is another important mechanism leading to cognitive decline. Brain energy metabolism heavily depends on blood supply, which provides oxygen and glucose to meet the metabolic needs of neurons. Duan [51] used non-invasive MRI to detect changes in cerebral blood flow and found that insufficient cerebral blood flow is closely associated with early cognitive decline in AD.

**Figure 5.**
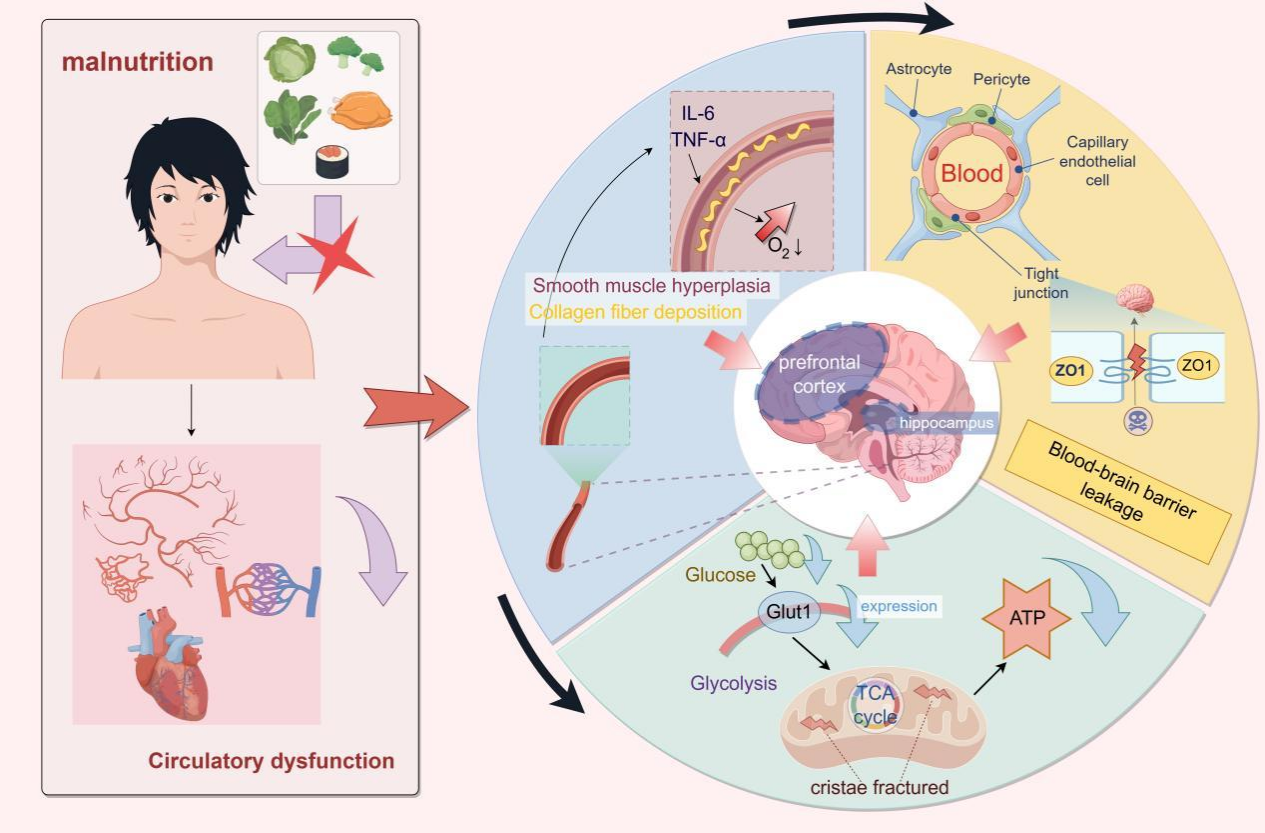
Malnutrition affects cognitive function through the cerebral blood supply pathway.

Abnormal cerebral hemodynamics is a core manifestation of insufficient cerebral blood flow. Malnutrition significantly affects cardiovascular function, leading to systemic circulation disorders and weakening the supply of cerebral blood flow. For example, anemia or low plasma protein levels are common in the elderly with disabilities. This condition reduces the oxygen-carrying capacity of red blood cells and blood volume, resulting in insufficient oxygen supply to the brain tissue [52]. Studies have shown that inadequate brain oxygen significantly weakens the function of energy-demanding brain areas such as the hippocampus and prefrontal cortex, thereby affecting memory, attention, and executive function [53]. Changes in cerebrovascular structure are also an essential consequence of malnutrition. Chronic malnutrition may induce abnormal proliferation of vascular smooth muscle cells and arteriosclerosis through the sustained elevation of pro-inflammatory factors (such as IL-6 and TNF-α), increasing cerebrovascular resistance [54]. This pathological vascular remodeling not only affects cerebral blood flow distribution but may also exacerbate the damage to brain tissue from toxic substances through BBB dysfunction [55] (according to Fig. 5).

### 3.7 Gut-Brain Axis Dysregulation

The gut-brain axis is a bidirectional network through which the gut and the central nervous system communicate and regulate each other via neural, immune, and metabolic pathways [56]. As a critical component of the gut-brain axis, the gut microbiome is vital in maintaining brain health and cognitive function.

Gut microbiome dysbiosis is a key link in the impact of malnutrition on the gut-brain axis. Kowalski [57] studied the association between gut-brain axis dysregulation and AD, demonstrating that gut microbiome dysbiosis promotes neuroinflammation and cognitive decline by inducing inflammation, increasing gut and blood-brain barrier permeability, and other mechanisms.

**Figure 6.**
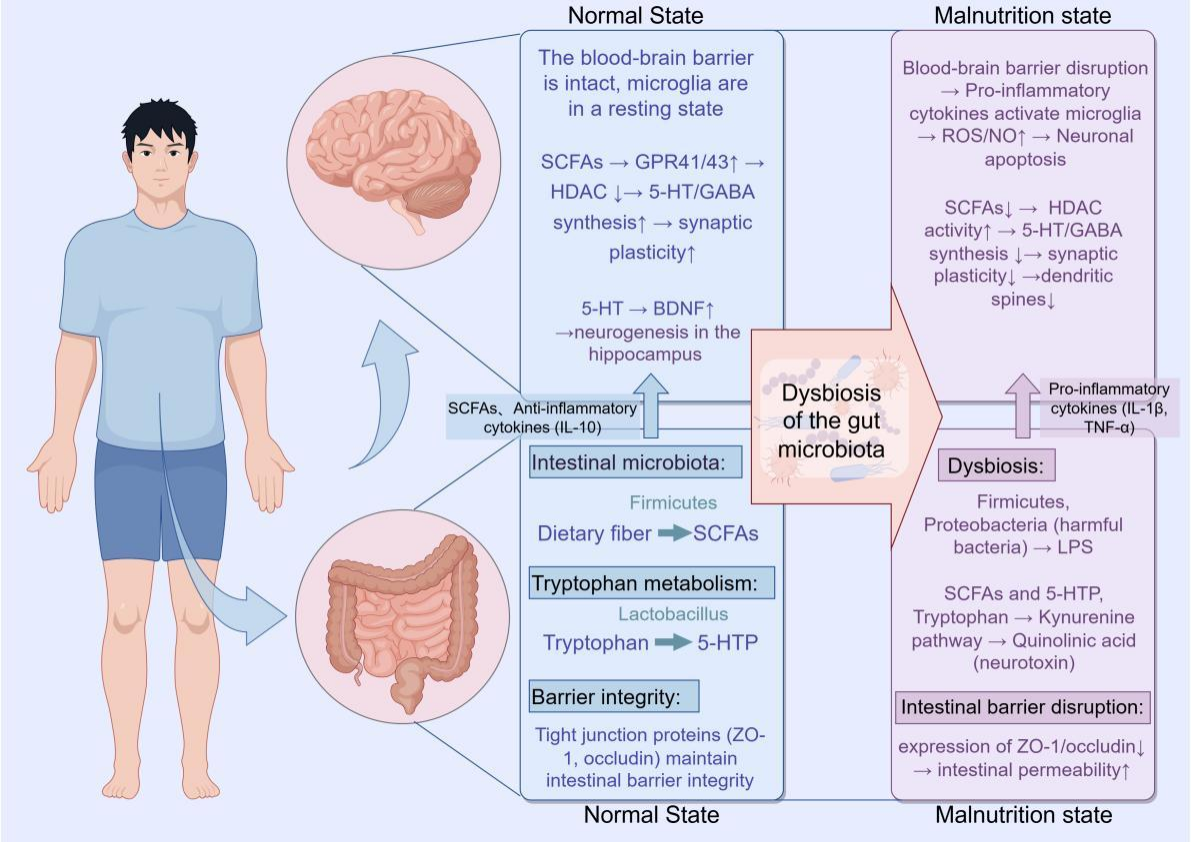
Malnutrition affects cognitive function through the gut-brain axis disorder pathway.

Changes in short-chain fatty acid (SCFA) levels are another essential feature of gut-brain axis dysregulation. SCFAs (such as acetate, propionate, and butyrate) are the primary products of gut microbial metabolism of dietary fiber, and they play an essential role in regulating neuroinflammation and maintaining the blood-brain barrier [58]. In a malnourished state, due to insufficient dietary fiber intake, the production of SCFAs significantly decreases, weakening their role in regulating neurotransmitter synthesis and inhibiting inflammatory factor production [59]. This may exacerbate neuroinflammation and synaptic dysfunction, further impairing cognitive abilities. Emerging research and mechanistic exploration suggest that the gut-brain axis influences cognitive function by regulating neurotransmitter synthesis and release. The gut microbiota can synthesize serotonin 5-hydroxytryptamine (5-HT) from tryptophan, a neurotransmitter crucial in regulating mood, memory, and cognition [60]. In a malnourished state, the gut microbiota’s ability to metabolize tryptophan decreases, potentially leading to insufficient brain 5-HT levels and further exacerbating cognitive decline [61] (according to Fig. 6).

In summary, malnutrition profoundly affects cognitive function through multiple mechanisms, including neurotransmitter synthesis disturbances, inadequate brain energy supply, chronic inflammation and oxidative stress, blood-brain barrier dysfunction, and gut-brain axis dysregulation. These mechanisms are intertwined and, from the molecular, cellular, and systemic levels, collectively accelerate neuronal damage and cognitive degeneration. Although research has revealed some of these mechanisms, the specific roles of different nutrients and the interactions between multiple mechanisms require further exploration. Therefore, we will focus on the critical role of specific nutrients in cognitive function protection, providing theoretical support for targeted nutritional interventions.

The molecular mechanisms are shown in Figure 1-6 (↑ or + indicates activation and increase in expression, ↓or - indicates inhibition and decrease in expression, and the other types of arrowheads indicate the modulatory effects), the above six images are created by Figdraw.

## 4. Association Between Specific Nutrients and Cognitive Function

### 4.1 The Relationship Between Protein Intake and Cognitive Function

Protein intake is closely related to cognitive function, particularly in older adults. Protein is a fundamental component required for neurotransmitter synthesis, and its deficiency can impair the production of neurotransmitters such as serotonin and dopamine, which in turn affects cognitive functions like mood, memory, and attention [62,63]. Moreover, insufficient protein intake may influence mental health through the "muscle-brain axis" mechanism. BDNF is secreted by skeletal muscles and plays a key protective role in synaptic plasticity and neuronal survival [64,65]. At the same time, a low-protein diet may weaken immune function, exacerbate chronic inflammation, and increase BBB permeability, allowing harmful substances to enter the brain and lead to neuronal damage [66].

Studies also suggest that the source of protein may affect its protective role in cognition. Due to their superior amino acid composition and higher bioavailability, animal-based proteins are considered more protective for cognitive function [67,68]. Although plant-based proteins may have insufficient amino acid compositions, they are rich in antioxidants and dietary fibers, which can support mental health through indirect mechanisms [69]. Therefore, individualized protein needs are significant for the elderly. Frail older adults should appropriately increase their intake of different protein sources to meet the body’s needs for repair and maintenance.

### 4.2 The Relationship Between Lipid Intake and Cognitive Function

Lipids are essential nutrients for maintaining brain structure and function, playing an important role in neuronal membrane stability, synaptic transmission, and inflammation regulation. Different types of lipids and their intake amounts may have significant effects on cognitive health. Studies suggest that specific types of fatty acids, such as Polyunsaturated fatty acids (PUFAs), particularly omega-3 fatty acids, are considered protective. In contrast, excessive intake of Saturated fatty acids (SFAs) and Trans fatty acids (TFAs) may increase the risk of cognitive decline and neurodegenerative diseases [70].

### 4.2.1 Saturated Fatty Acids and Cognitive Decline

SFAs are widely present in animal fats, tropical plant oils, and processed foods. Excessive intake of SFAs is significantly associated with cognitive decline and an increased risk of AD [71]. Studies indicate that high SFA diets may negatively affect brain health through several mechanisms. First, excessive SFA intake induces insulin resistance, weakening the brain cells' ability to utilize glucose and leading to disrupted energy metabolism [72]. Second, high SFA intake activates inflammatory pathways, increasing the release of pro-inflammatory factors (such as TNF-α and IL-6). These factors act on the brain via the BBB, triggering neuroinflammation and worsening neuronal dysfunction [73].

Furthermore, SFA metabolism may cause mitochondrial dysfunction and endoplasmic reticulum stress, leading to insufficient neuronal energy supply and cell apoptosis [74]. SFAs may also promote the pathogenesis of AD by increasing β-amyloid protein (Aβ) deposition. Studies have shown that high SFA diets accelerate Aβ formation and reduce its clearance efficiency, further aggravating pathological changes in the brain and impairing cognitive function [75].

### 4.2.2 Trans Fatty Acids and Cognitive Decline

TFAs are primarily found in partially hydrogenated vegetable oils and processed foods. Studies have shown that compared to cis fatty acids, trans fatty acids increase the amyloidogenic processing of Amyloid precursor protein (APP) in AD and reduce non-amyloidogenic processing. This leads to an increased production of Aβ peptides, thereby increasing the risk of AD and accelerating cognitive decline [76]. Research by Tan et al. [77] suggests that trans fatty acids promote the entry of immune cells and cytokines into the brain, inducing local inflammation and increasing oxidative stress, further accelerates the development of cognitive dysfunction.

### 4.2.3 Polyunsaturated Fatty Acids PUFAs and the Protective Role in Cognitive Function

PUFAs are essential fatty acids, meaning the human body cannot synthesize them independently and must obtain them through diet. PUFAs are crucial in human health, particularly in the brain, cardiovascular system, and immune function [78]. PUFAs mainly include omega-3 fatty acids and omega-6 fatty acids. Omega-3 fatty acids primarily come from deep-sea fish (such as salmon and sardines) and have anti-inflammatory effects, promote brain function development, and protect cardiovascular health [79]. Omega-6 fatty acids primarily come from vegetable oils and nuts, play a role in inflammatory responses, and support immune function, but excessive intake may promote chronic inflammation [80].

PUFAs, particularly Docosahexaenoic acid (DHA) and Eicosapentaenoic acid (EPA) in omega-3 fatty acids, are essential components of neuronal membranes. Sufficient intake of PUFAs can maintain the fluidity of neuronal membranes and synaptic plasticity, thereby enhancing the efficiency of synaptic signaling [81,82]. DHA promotes the expression of BDNF and reduces the production of pro-inflammatory factors (such as TNF-α and IL-6) by regulating inflammatory pathways. In AD and other neurodegenerative diseases, inflammation is a key mechanism leading to cognitive decline, and supplementation with EPA and DHA has been shown to slow down this process effectively [83]. Additionally, the antioxidant properties of PUFAs can reduce free radical production and protect neurons from oxidative stress damage [84].

Although PUFAs have significant neuroprotective effects, the ratio of their intake is crucial for health. A high intake of omega-6 fatty acids may promote chronic inflammation, whereas omega-3 fatty acids can counteract this inflammatory effect [85]. Studies suggest that appropriately increasing the ratio of omega-3 fatty acids in the diet while controlling the intake of omega-6 fatty acids can more effectively improve brain health and protect cognitive function [86].

Therefore, the quality and quantity of lipid intake have an essential impact on the cognitive function of frail elderly individuals. Properly adjusting the intake structure of dietary fatty acids—reducing the intake of SFA and TFA while increasing PUFA, especially omega-3 fatty acids—can help prevent or delay cognitive decline.

### 4.3 The Association Between Carbohydrate Intake and Cognitive Function

Carbohydrates are the main energy source for the human body and brain, and the quantity and quality of carbohydrate intake significantly affect cognitive function. The metabolic product of carbohydrates, glucose, provides energy support to neurons, but the effects of different types and sources of carbohydrates on brain health vary significantly. The intake of high-quality, low Glycemic index (GI) carbohydrates helps maintain cognitive function. In contrast, a long-term high-sugar diet or the intake of low-quality carbohydrates may increase the risk of cognitive decline and neurodegenerative diseases [87, 88].

Carbohydrates' impact on cognitive function is mainly mediated through regulating blood sugar levels, insulin sensitivity, and inflammation. Excessive intake of high-GI carbohydrates leads to larger fluctuations in blood sugar levels, worsening insulin resistance and inflammatory responses, negatively affecting brain health [89]. In contrast, a low-GI diet provides a stable glucose supply, optimizes brain energy metabolism, and reduces oxidative stress and neuroinflammation [90].

### 4.3.1 High-Sugar Diet and Cognitive Decline

High-sugar diets accelerate cognitive decline through multiple mechanisms. Excessive sugar intake leads to larger fluctuations in blood glucose levels, triggers insulin resistance, weakens glucose utilization in the brain, and causes disruptions in neuronal energy metabolism [91]. A prolonged high blood sugar state has been confirmed to be closely associated with cognitive decline and an increased risk of AD [92]. Furthermore, a high-sugar diet promotes the formation of Advanced glycation end-products (AGEs), which accumulate in the brain and activate inflammatory responses, impair synaptic function, and increase Aβ deposition, worsening cognitive degeneration [93,94]. At the same time, high-sugar diets significantly raise pro-inflammatory factors, activate inflammatory pathways, further damage the blood-brain barrier, and impair synaptic plasticity [95].

### 4.3.2 Dietary Fiber and Cognitive Decline

Dietary fiber is a special type of carbohydrate that does not directly provide energy but has far-reaching effects on human health. Epidemiological studies show that insufficient dietary fiber intake is closely related to cognitive decline. At the same time, fiber-rich diets (such as the Mediterranean and high-fiber diets) are associated with better mental performance. This effect is mainly mediated by improving gut microbiota, reducing systemic inflammation, and regulating metabolic states to positively affect cognition.

One important mechanism by which dietary fiber protects cognitive function is regulating gut microbiota. Microbes in the gut ferment dietary fiber to produce SCFAs, such as butyrate, propionate, and acetate. These metabolites provide energy for gut epithelial cells and enter the brain via the bloodstream, exerting anti-inflammatory and neuroprotective effects [96,97]. Studies show that butyrate can regulate the expression of BDNF, promoting synaptic plasticity and thus improving learning and memory ability [98].

Another key pathway through which dietary fiber benefits cognitive health is its anti-inflammatory and immune-modulating effects. Dietary fiber can reduce pro-inflammatory factors and alleviate chronic inflammation [99]. In addition, it can balance the gut microbiota, reduce intestinal permeability, and lower the production of toxic metabolites, thereby decreasing the risk of inflammatory mediators entering the bloodstream and indirectly protecting the blood-brain barrier [100,101].

Thus, a balanced intake of carbohydrates, with a proper ratio of simple and complex carbohydrates, is key to maintaining health and efficient energy metabolism. By prioritizing fiber-rich whole grains, vegetables, and fruits while limiting refined sugar intake, one can obtain the best health benefits from carbohydrates.

### 4.4 The Relationship Between Vitamin Intake and Cognitive Function

Vitamins are essential nutrients for maintaining normal nervous system function. Different types of vitamins play unique roles in antioxidation, neuroprotection, and metabolic regulation, making them crucial for preventing cognitive decline and neurodegenerative diseases.

Fat-soluble vitamins (such as vitamins A, D, and E) are key in protecting brain tissue from oxidative stress damage [102]. Vitamin A participates in antioxidation and supports neuronal development and synaptic function by regulating the retinoic acid receptor signaling pathway [103,104]. Studies have shown that vitamin A deficiency may lead to increased neuroinflammation and is closely associated with cognitive decline [105]. Vitamin D also plays a crucial role in neuronal survival and synaptic function. Its deficiency has been linked to increased risks of chronic inflammation, neuroinflammation, and neurodegenerative diseases [106]. A study by Jiang [107] found that vitamin D can inhibit neuroinflammation by regulating the TLR4/MyD88/NF-κB signaling pathway, thereby reducing brain edema, BBB disruption, and cognitive impairment caused by traumatic brain injury. Vitamin E is the brain’s primary fat-soluble antioxidant, protecting the integrity of neuronal membranes by neutralizing free radicals [108]. In particular, studies on AD models have shown that vitamin E can reduce Aβ deposition and Tau protein hyperphosphorylation while increasing the expression of BDNF [109]. Research indicates that individuals with higher vitamin E levels perform better on memory and cognitive tests, whereas vitamin E deficiency may increase the risk of AD [110].

Water-soluble vitamins (such as B vitamins and vitamin C) play a crucial role in neurotransmitter synthesis and energy metabolism [111,112]. B vitamins (such as B6, B12, and folic acid) are key factors in homocysteine metabolism, and elevated homocysteine levels have been proven to be associated with cognitive decline [113]. Supplementing B vitamins can help lower homocysteine levels, improve cerebral blood flow, and thereby slow cognitive deterioration [114]. Vitamin C, as a water-soluble antioxidant, helps reduce free radical production and protects the collagen structure in the brain, thereby supporting the integrity of the BBB and promoting synapse formation [115,116].

Moreover, different vitamins may exert synergistic effects to enhance their protective impact on cognitive function further. For example, combined supplementation of vitamins E and C has been shown to significantly reduce oxidative stress markers [117]. In contrast, the joint intake of B vitamins and vitamin D can enhance the suppression of neuroinflammation [118].

Therefore, adequate vitamin intake is essential for protecting cognitive function and preventing neurodegenerative diseases. Long-term vitamin deficiency increases the risk of cognitive impairment. In contrast, a balanced diet or appropriate vitamin supplementation can effectively slow age-related mental decline and reduce the likelihood of developing neurodegenerative conditions.

### 4.5 The Relationship Between Mineral Intake and Cognitive Function

Minerals are essential micronutrients for the human body and are categorized into macrominerals (such as calcium, magnesium, and potassium) and trace minerals (such as iron, zinc, and selenium). They are crucial in supporting the nervous system function, maintaining metabolic balance, and preventing various diseases.

Calcium, primarily sourced from dairy products and leafy green vegetables, has been linked to a reduced risk of cognitive decline. A study by Fleet [119] suggested that insufficient calcium absorption may indirectly affect neurotransmitter synthesis and cognitive function. In a cohort study on AD, low serum calcium levels were associated with an increased risk of Mild cognitive impairment (MCI) progressing to early AD, indicating that calcium levels may be an essential factor influencing cognitive decline [120]. Another study found that low calcium intake could elevate Parathyroid hormone (PTH) levels and intracellular calcium in vascular smooth muscle cells, leading to vasoconstriction and affecting cognitive health [121].

Magnesium, found in leafy green vegetables, nuts, whole grains, and legumes, has been shown to correlate with better performance in attention, executive function, and language ability tests among middle-aged and older adults. Magnesium levels are significantly negatively associated with MCI, suggesting that magnesium may be a key nutrient in maintaining cognitive function [122]. Kumar et al. explored magnesium’s critical role in neuronal health, including its regulation of ion channels, synaptic plasticity, and neurotransmitter release. Magnesium also protects neurons from oxidative stress and excitotoxicity, enhancing cognitive function [123].

Sodium is primarily sourced from table salt. Studies have shown that a high-sodium diet may be associated with poorer cognitive performance, as excessive sodium intake has been linked to cognitive impairment and an increased risk of dementia, suggesting that reducing sodium intake could be a potential preventive strategy [124]. Research by Yuan et al. [125] indicated that a high-salt diet in elderly mice disrupted the Tricarboxylic acid (TCA) cycle, induced excessive tau protein phosphorylation, and impaired synaptic function, ultimately leading to cognitive decline. Another study [126] found that a high-salt diet in mouse models resulted in impaired learning and memory abilities, along with gut microbiota dysbiosis, reduced SCFAs, BBB damage, neuroinflammation, and hippocampal neuron apoptosis. These mechanisms may explain the negative effects of excessive salt intake on cognitive function.

Zinc is mainly found in seafood, such as oysters and crabs. Research has shown an L-shaped relationship between dietary zinc intake and cognitive decline in the elderly, with a daily intake of 8.8 mg of zinc significantly slowing the rate of cognitive decline. This association is particularly pronounced in individuals with lower levels of physical activity [127]. Wu et al. [128] demonstrated in mouse experiments that zinc deficiency leads to Golgi apparatus dysfunction and amyloid protein accumulation, changes closely related to hippocampal neuron dysfunction and cognitive impairment. Sun et al. [129] explored the role of zinc and its associated proteins in cognitive impairment and aging, summarizing the molecular mechanisms of zinc in regulating neuronal metabolism and synaptic plasticity. Their findings suggest that zinc deficiency may weaken synaptic function, leading to cognitive decline.

Selenium is primarily found in Brazil nuts, seafood, and eggs. Studies have shown that selenium supplementation significantly improves selenium levels and cognitive test performance in individuals with MCI and AD, with even greater benefits when combined with other nutrients [130]. The underlying mechanism may involve the neuroprotective effects of selenoproteins through their antioxidant and anti-inflammatory properties [131]. However, excessive selenium intake may lead to neurological dysfunction, highlighting the importance of balanced selenium consumption. A recent study by Lv [132] indicated that selenium deficiency affects hippocampal myelination and lipid metabolism through selenoprotein H regulation, ultimately leading to cognitive decline. This mechanism provides new insights into selenium’s role in neurodegenerative diseases.

Copper is mainly sourced from liver and oysters. A study investigating the nonlinear effects of copper intake on cognitive function in older adults found that copper intake below 0.8 mg/day was positively associated with mental performance. In contrast, excessive intake may be detrimental [133]. The underlying mechanism may involve impaired synaptic plasticity and inhibition of the CREB/BDNF signaling pathway [134]. A study by Lamtai [135] demonstrated that prolonged copper exposure induces oxidative stress, impairing hippocampal function in rats and leading to declining learning and memory abilities. Significant changes in oxidative stress markers, such as superoxide dismutase and catalase, confirmed the neurotoxic effects of copper.

Iodine is primarily found in seaweed and marine fish. A study on adult iodine deficiency indicated that insufficient iodine intake is associated with cognitive impairment and thyroid dysfunction [136]. Research by Aceves [137] suggested that molecular iodine exerts positive effects on brain function through its antioxidant and immune-modulating properties, with potential benefits for neuronal metabolism and inflammation regulation. However, most studies on iodine intake have focused on its impact during early developmental stages, confirming its crucial role in brain development, particularly in neuronal differentiation, synapse formation, and neurogenesis during specific critical periods [138].

Thus, minerals support nervous system function and cognitive health through multiple mechanisms. A balanced diet is the best way to ensure adequate mineral intake, emphasizing whole grains, leafy greens, nuts, dairy products, and seafood while avoiding excessive mineral consumption. This approach promotes neuronal health and effectively helps prevent cognitive decline.

### 4.6 The Relationship Between Polyphenols and Cognitive Function

Polyphenols are a group of naturally occurring compounds found in plants, known for their potent antioxidant and anti-inflammatory properties. Research suggests that polyphenols significantly protect adult cognitive function, particularly in slowing cognitive decline and preventing neurodegenerative diseases such as AD. These protective effects are primarily mediated through improved cerebral blood flow, neurotransmitter regulation, and antioxidant and anti-inflammatory mechanisms.

### 4.6.1 Flavonoids and Cognitive Decline

Flavonoids are widely present in plant-based foods, and higher dietary intake of total flavonoids has been associated with better cognitive health. Specific flavonoid subclasses, such as flavan-3-ols, catechins, and anthocyanins, have shown a strong correlation with cognitive function, likely due to their antioxidant, anti-inflammatory, and neuroprotective properties [139]. Research by Bakoyiannis et al. [140] has identified several mechanisms by which flavonoids improve cognition, including activating the ERK/CREB/BDNF and PI3K/Akt signaling pathways to promote neuronal proliferation, reducing oxidative stress and enhancing synaptic plasticity. A recent study also found that flavonoids could improve cognitive function through the gut-brain axis, with mechanisms involving gut microbiota regulation, reduction of neuroinflammation, and clearance of neurotoxic proteins [141].

### 4.6.2 Anthocyanins and Cognitive Decline

Anthocyanins, natural antioxidants, are primarily found in blueberries and blackberries. Studies have explored the relationship between anthocyanin intake and memory performance in older adults with MCI. Findings suggest that participants with higher anthocyanin intake performed better in both short-term and long-term memory tests, indicating a potential role of anthocyanins in memory preservation [142]. Ellis et al. [143] used Functional magnetic resonance imaging (fMRI) to examine the impact of anthocyanin intake on cognitive function, revealing that anthocyanins enhance verbal memory and working memory, as well as increase cerebral blood flow. Vauzour et al. [144] investigated the mechanisms by which anthocyanins improve learning and cognitive abilities in aging animal models, reporting that anthocyanins enhance the expression of key synaptic proteins while reducing levels of apoptosis-related proteins.

Although the neuroprotective effects of polyphenols and their underlying mechanisms have been extensively studied, practical applications still face challenges. For instance, the low bioavailability of polyphenols may limit their effective concentration in the brain, necessitating further optimization of polyphenol formulation and delivery systems. Additionally, the synergistic effects of polyphenols and personalized intake strategies require further exploration.

## 5. Practical Applications for Dietary Interventions in the Prevention and Management of Cognitive Impairment

In recent years, the role of dietary interventions in the prevention and management of cognitive impairment has garnered significant attention. The European Society for Clinical Nutrition and Metabolism (ESPEN) in 2023 updated guidelines on nutrition in critically ill patients, emphasizes the need for precision nutrition interventions based on an individual's skeletal muscle mass, metabolic status, and inflammatory levels [145]. The guidelines recommend prioritizing enteral nutrition or oral intake to meet energy demands while gradually adjusting to target intake levels to mitigate the risk of refeeding syndrome.For populations at high risk of cognitive decline, ESPEN further advocates a dual-strategy approach: optimizing protein intake (≥1.2 g/kg/day) and supplementing with omega-3 fatty acids (e.g., 2 g of EPA/DHA per day). Studies have demonstrated that this regimen can mitigate neuroinflammation by inhibiting the NF-κB signaling pathway, thereby improving executive function in patients with MCI [146].

Several studies and authoritative reports have underscored the strong association between metabolic syndrome (including hypertension and diabetes) and cognitive decline. As a preventive measure, they recommend adopting dietary patterns such as the Mediterranean Diet (MD) or the Dietary Approaches to Stop Hypertension (DASH) diet while strictly limiting the intake of ultra-processed foods (e.g., sugar-sweetened beverages and pre-packaged snacks)[147,148].A recent longitudinal study analyzed 30 years of dietary data from participants and assessed their health status after the age of 70, incorporating key indicators such as cognitive function, physical capability, mental health, and the presence of major chronic diseases (e.g., cardiovascular disease, cancer, and type 2 diabetes). The findings revealed that individuals with long-term high consumption of ultra-processed foods exhibited a significantly lower likelihood of achieving healthy aging [149]. Numerous studies have linked these foods to cognitive decline and hippocampal atrophy, highlighting their detrimental impact on brain health [150,151].

The MD, centered around olive oil, deep-sea fish, and antioxidant-rich fruits and vegetables, is rich in polyphenolic compounds that can promote the expression of BDNF. This diet also modulates the gut microbiota, such as increasing the abundance of butyrate-producing bacteria, thereby reducing the risk of Aβ deposition [152,153]. A cohort study explored the relationship between adherence to a Western-adapted Mediterranean diet and changes in episodic memory over a nine-year period in an adult sample. The study found that long-term adherence to the Mediterranean diet was associated with better cognitive health, especially in terms of episodic memory [154]. This finding supports the inclusion of the Mediterranean diet as a key strategy in cognitive health interventions.

The DASH Diet emphasizes a high intake of potassium, fiber, and low sodium, which may reduce the risk of mental impairment by improving vascular endothelial function [155]. A study following 923 middle-aged and older participants revealed that high adherence to the DASH diet was linked to a lower incidence of AD [156]. Notably, the MIND Diet (Mediterranean-DASH Diet Intervention for Neurodegenerative Delay), which combines the benefits of both the Mediterranean and DASH diets, has shown even greater efficacy in reducing the incidence of AD [157,158]. A prospective cohort study involving 16,058 women aged 70 and older found that long-term adherence to the MIND diet was moderately associated with better verbal memory in later life [159].

Dietary interventions have been shown to improve cognitive function in older adults with disabilities by modulating the gut microbiota. Probiotics and prebiotics are crucial in the gut-brain axis [160,161]. Studies have demonstrated that supplementation with Lactobacillus and Bifidobacterium strains can significantly enhance cognitive scores in patients with AD [162]. Meanwhile, prebiotics such as fructooligosaccharides facilitate the production of SCFAs, which help strengthen the BBB and reduce neuroinflammation [163]. MD, rich in dietary fiber, antioxidants, and PUFAs, has been found to promote the growth of beneficial gut bacteria while reducing the abundance of pro-inflammatory microbial populations. This enhancement in gut microbiota diversity is positively correlated with improvements in cognitive function [164,165].

A growing body of high-quality research supports the critical role of dietary interventions in the prevention and management of cognitive impairment. In particular, the Mediterranean Diet, DASH Diet, and MIND Diet have demonstrated significant potential in mitigating cognitive decline and lowering the risk of AD through distinct biological pathways.

## 6. Conclusion and Future Prospects

The relationship between malnutrition and cognitive decline in disabled elderly individuals is a topic of significant practical importance and challenge in aging health research. By systematically reviewing existing literature, this paper summarizes the key mechanisms through which malnutrition accelerates cognitive deterioration. These mechanisms include neuro-transmitter synthesis disorders, brain energy metabolism imbalances, chronic inflammation and oxidative stress responses, blood-brain barrier dysfunction, reduced neuroplasticity, and gut-brain axis dysregulation. Meanwhile, this study provides an in-depth exploration of the multifaceted regulatory effects of specific nutrients—such as proteins, fatty acids, vitamins, and minerals—on cognitive health. It further elucidates their potential protective mechanisms in mitigating cognitive decline and highlights the critical role of dietary interventions in the prevention and management of cognitive disorders. These findings not only deepen our understanding of the health status of disabled elderly individuals but also provide a scientific basis for implementing nutritional intervention programs.

Despite the wealth of data and theoretical support provided by existing studies on the relationship between malnutrition and cognitive decline, several scientific gaps and research limitations remain. Firstly, the combined effects of different nutrients and their interactions with individual genetic and metabolic characteristics are not yet fully understood. Since the nutritional needs of elderly populations vary significantly, developing precise and personalized intervention strategies remains a critical research direction. Secondly, most current studies rely on cross-sectional or observational research, lacking high-quality longitudinal studies and randomized controlled trials (RCTs) to establish causal relationships and validate intervention outcomes. Furthermore, there is still insufficient understanding of how malnutrition interacts with cognitive function via the gut-brain axis, particularly regarding how gut microbial metabolites influence brain health through immune, inflammatory, and neuro-transmitter regulatory pathways.

Future research should integrate multi-omics technologies (such as genomics, metabolomics, and gut microbiome analysis) and biomarker studies to construct a comprehensive network linking malnutrition and cognitive decline. Large-scale, long-term cohort studies and multi-center randomized controlled trials will be essential for verifying existing theoretical hypotheses and uncovering the unique roles of various nutrients or dietary patterns in cognitive function preservation.

In summary, the relationship between malnutrition and cognitive decline is multifaceted and complex. Its study and practical applications hold not only theoretical value but also significant implications for health equity and sustainable social development in the context of global aging. With the dual drive of scientific research and social practice, nutritional intervention programs are expected to make groundbreaking progress in improving cognitive health and quality of life among disabled elderly individuals. Additionally, these advancements could alleviate the long-term caregiving burden on families and society, contributing to the realization of the goal of healthy aging.
